# Effects of the carrier frequency of interferential current on pain modulation in patients with chronic nonspecific low back pain: a protocol of a randomised controlled trial

**DOI:** 10.1186/1471-2474-14-195

**Published:** 2013-06-27

**Authors:** Juliana Barbosa Corrêa, Leonardo Oliveira Pena Costa, Naiane Teixeira Bastos de Oliveira, Kathleen A Sluka, Richard Eloin Liebano

**Affiliations:** 1 Master’s and Doctoral Programs in Physical Therapy, Universidade Cidade de São Paulo (UNICID), São Paulo, Brazil; 2 Graduate Program in Physical Therapy and Rehabilitation Science, University of Iowa, College of Medicine, Iowa City, IA, USA

**Keywords:** Low Back Pain, Electric Stimulation, Central Nervous System Sensitization, Central Sensitization, Physical Therapy Modalities

## Abstract

**Background:**

Low back pain is an important public health problem that is associated with poor quality of life and disability. Among the electrophysical treatments, interferential current (IFC) has not been studied in patients with low back pain in a high-quality randomised controlled trial examining not only pain, but pain mechanisms and function.

**Methods/design:**

A three-arm randomised controlled trial with patient and assessor blinded to the group allocation. One hundred fifty patients with chronic, nonspecific low back pain from outpatient physical therapy clinics in Brazil. The patients will be randomly allocated into 3 groups (IFC 1 kHz, IFC 4 kHz or Placebo IFC). The interferential current will be applied three days per week (30 minutes per session) over four weeks. *Primary outcome*: Pain intensity. *Secondary outcomes*: The pressure pain threshold, global impression of recovery, disability, function, conditioned pain modulation and temporal summation of pain, discomfort caused by the current. All outcomes will be measured at 4 weeks and 4 months after randomisation. The between-group differences will be calculated by using linear mixed models and Tukey’s post-hoc tests.

**Discussion:**

The use of a placebo group and double-blinding assessor and patients strengthen this study. The present study is the first to compare different IFC carrier frequencies in patients with chronic low back pain.

**Trial registration:**

Brazilian Registry of Clinical Trials: http://RBR-8n4hg2

## Background

Low back pain is an important public health issue that directly affects an individual’s quality of life and activities of daily living (ADLs) [1]. Low back pain is also responsible for many absences from work and has high worldwide socioeconomic costs [2]. The prognosis with acute and persistent low back pain is favourable, and the condition improves or resolves in the majority within the first 6 weeks. However, low to moderate pain levels and disability can persist in patients who become chronic [3].

Inefficient endogenous pain control and central sensitivity are important characteristics in patients with low back pain [4]. Prolonged afferent nociceptive impulses may lead to increased excitability of the central sensory neurons and changes in their plasticity that lead to hypersensitivity resulting in an exaggerated response to pain [5]. Increasing evidence supports the clinical significance of the central sensitisation in patients with unexplained chronic pain; therefore, reduction in central sensitisation should be targeted for the treatment of these patients. The use of conservative therapies such as transcutaneous electrical nerve stimulation (TENS) and manual therapy in experimental models suggests that these treatments can reduce the central sensitization in animals and could desensitise the central nervous system (CNS) in humans [6,7].

The treatment of chronic low back pain aims to reduce pain and disability [8]. Exercise is widely prescribed for treating patients with chronic low back pain [9]. However, patients with low back pain may have significant pain that limits their physical capacity, making it difficult to exercise [10]. Therefore, the use of electrophysical agents to decrease pain could enable these patients to participate in an exercise program at an earlier stage of recovery [10]. Among the electrotherapeutical resources, interferential current (IFC) has been studied for the treatment of acute [11] and chronic low back pain [10]. A recent systematic review concluded that when combined with other treatments, such as exercises and massage, IFC demonstrates advantages over placebo and non-treatment control groups in reducing the intensity of pain associated with musculoskeletal disorders [12]. However, little evidence has indicated that the use of IFC alone can reduce the intensity of pain [12], disability or use of analgesics, or improve function in patients with chronic low back pain [10].

Despite various adjustments to the amplitude-modulated frequency (AMF) often used in clinical practice to treat different injury or disease stages, studies have indicated that AMF does not influence hypoalgesia in healthy individuals [13,14], which suggests that the main parameter that should be adjusted is the carrier frequency of the current to affect the pain inhibitory mechanisms [13]. A 2 kHz carrier frequency is often used to strengthen the muscles, and a 4 kHz frequency is used to produce analgesia. However, this conduct is based on therapists’ personal observations, equipment manuals [15] and not on controlled studies. Only one study [16] has compared the effect of the carrier frequencies of the interferential current on the pressure pain threshold in healthy individuals. This study demonstrated that a 1 kHz frequency provides a higher hypoalgesic response compared with 8 kHz or 10 kHz during and after IFC stimulation. However, the evidence on the use of IFC alone for decreasing pain remains insufficient. In addition, chronic low back pain appears to be linked to central sensitisation and a deficiency in the activation of the central pain inhibition mechanisms. Thus, these patients could possibly benefit from the use of IFC to relieve their symptoms. For these reasons, we decided to conduct a prospective randomized controlled study to assess the effects of IFC on pain at rest and during movement, and the disability in patients with chronic low back pain. This study also aims to assess if the use of IFC would reactivate the innate mechanism of conditioned pain modulation and decrease the central hypersensitivity in chronic low back pain patients.

## Methods /design

### Study design

**Figure 1:**
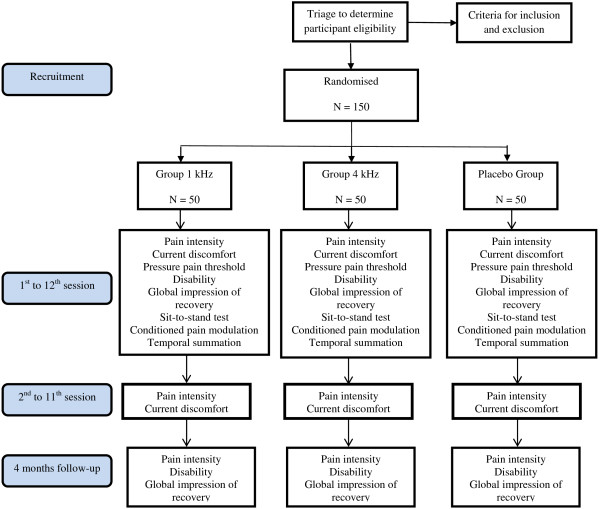
Flowchart of the study.

This is a three-arm randomised controlled trial with patient and assessor blinded to the group allocation. Figure 1 provides a flowchart of the study.

### Participants, therapists, centres

Patients seeking physical therapy treatment at the clinic of the Universidade Cidade de São Paulo - UNICID and the Centro de Especialidades Médicas de Guarulhos - CEMEG through medical referral who present nonspecific low back pain for at least 3 months and with a minimum pain intensity of 3 in the 0–10 pain numeric rating scale (NRS-Brazilian-Portuguese version) [17] during the last 7 days will be included in the study. Patients of both genders with ages ranging from 18 to 80 years old will be included. Patients with severe spinal disorders such as fractures, tumours and inflammatory diseases including ankylosing spondylitis; patients with nerve root disorders confirmed by neurological exams (herniated disks and spondylolisthesis with neurological involvement, spinal canal stenosis and others); patients suffering from neurological diseases, severe cardiopulmonary diseases, pregnancy, infection or skin lesions at the current application site, cancer, or changes in sensitivity and allergy in the region of electrode positioning; and patients who require artificial cardiac pacemakers will be excluded.

Participants will be assessed by the researcher responsible for the assessments during the study to verify that they fit the inclusion criteria. If eligible, they will be informed of the study objectives. They will then sign an informed consent form to participate in the study. The present study was approved by the Research Ethics Committees of Universidade Cidade de São Paulo and Centro de Especialidades Médicas de Guarulhos.

Due to the nature of the intervention, only the therapist responsible for application of IFC will be aware about group allocation; the assessor and the participants will be blinded to the group allocation. To keep the evaluator blind to the study groups, the device will be covered with a dark cloth.

### Intervention

Each participant will be randomly allocated to one of 3 groups: Group IFC 1 kHz (n = 50), Group IFC 4 kHz (n = 50) and a Placebo IFC Group (n = 50). All participants will receive 30 minutes of current stimulation (the current amplitude will not be increased for the participants in the placebo group) 3 times per week on alternate days for 4 weeks for a total of 12 sessions.

The present study will use the Neurovector equipment that produces a medium-frequency alternating current (Neurovector, Indústria Brasileira de Equipamentos Médicos - IBRAMED®, Amparo, Brazil). This equipment was developed exclusively for the present study and is not commercially available. The technique used will involve a bipolar mode with 2 channels located 5 cm from the L3 and L5 spinous processes. The following parameters will be employed: current carrier frequency according to the patient group (1 or 4 kHz); AMF = 100 Hz; Sweep = 50 Hz; 1:1 swing pattern and 30 minutes of stimulation.

The procedures for the placebo group will be similar to those of the other groups; however, the current amplitude will not be increased. The patients will be informed that they may or may not feel any sensation at the application site of the electrodes [13]. For the active groups, the therapist will increase the current amplitude until the participant reports feeling a strong but comfortable tingling. Every 5 minutes, the therapist will ask each participant whether the “strong but comfortable tingling” remains. In case of sensory habituation, the current amplitude will be increased until the participant reaches the previous sensation. After ending the application, the patient will wait for 20 minutes for the necessary measurements be taken.

### Outcome measures

#### Primary outcome

Pain intensity after all treatment sessions and 4 months after randomisation.

#### Secondary outcomes

(1) pressure pain threshold; (2) current discomfort; (3) disability; (4) global impression of recovery; (5) functional performance; (6) conditioned pain modulation and pain temporal summation; and (7) analgesic use measured in all time-points.

### Measurement instruments

#### Pain numerical rating scale (NRS)

Pain intensity will be assessed using the pain numerical rating scale (NRS) [18], which assesses the level of pain intensity perceived by the patient through an 11-point scale (ranging from 0 to 10), with 0 indicating “no pain” and 10 indicating the “worst possible pain”. The pain assessment will be carried out verbally with the patient reporting the pain intensity. This instrument has been translated and cross-culturally adapted for the Brazilian population [17].

The pain will be assessed prior to applying the current, at 30 minutes of treatment with the current still on and 20 minutes after the current is turned off. This variable will be measured in all sessions, after treatment and in the follow-up examination after 4 months.

#### Pressure pain threshold (PPT)

The pressure pain threshold (PPT) will be measured using a Somedic digital pressure algometer (Somedic Inc., Hörby, Sweden). Assessments will be performed prior to application of the current, immediately after the 30^th^ minute of stimulation and 20 minutes after the session has ended. This assessment will be performed in the first and last sessions.

Hygienisation with soap and water will be performed at the assessed sites. After cleaning the skin, the areas to which the algometer will be attached will be marked with a tape measure and a pen. Two points will be marked bilaterally: the first located 5 cm lateral to the L3 spinous process [19] and the second located 5 cm lateral to the L5 spinous process [20]. A point, to be used as a control, will also be marked on the tibialis anterior muscle of the right leg, 5 cm lateral to the tibial tuberosity [21].

The assessor will conduct a preliminary study of intra-observer reliability for measuring the pressure pain threshold at the evaluation points that will be used in the study. Ten participants with chronic low back pain will be recruited and assessed on two occasions that are 48 hours apart. The intra-evaluator reliability for the measurement of the PPT will be estimated by calculating the intraclass correlation coefficients (ICC type 3,2) for the tibialis anterior muscle and the low back muscles.

During the PPT measurements, the circular probe of the algometer (1-cm^2^ area) will be positioned perpendicular to the skin and pressed at approximately 50 kPa/s [22]. Participants will be asked to press a button when the pressure or discomfort sensation becomes clearly painful. Two pressure measurements (in kPa) will be collected from each area at 30-s intervals.

Two demonstrations of the procedure will be conducted for each participant in the extensor muscles of the dominant forearm to ensure that the test is well-understood. The mean values will be used for the lumbar region and the tibialis anterior muscle in the pain threshold data analysis.

#### Current discomfort

The discomfort caused by the current will be assessed using a 10-cm visual analogue scale (VAS) where the far left end indicate “very comfortable” and the far right end indicate “very uncomfortable” [23]. The discomfort will be assessed at 30 minutes of stimulation in all sessions.

#### Roland-Morris disability questionnaire

Disability will be assessed by the Roland-Morris disability questionnaire, which is widely used to assess the functional performance associated with low back pain [24]. This instrument, which has been translated and cross-culturally adapted for the Brazilian population [25], consists of 24 items that describe the daily activities that prove difficult for the patients to perform due to low back pain. Many of the selected items are directly correlated to a greater impairment in functional performance. The patients will be instructed to verbally state which items describe them on the particular assessment day. The questionnaire will be applied on the first and last days of treatment and at the 4-month follow-up (conducted by telephone).

#### Global perceived effect scale

The global impression of recovery will be assessed through the global perceived effect scale [26,27], which has been translated and cross-culturally adapted into Brazilian-Portuguese [17] and corresponds to an 11-point scale ranging from −5 points (much worse) to 0 (no change) to 5 points (completely recovered). To measure the global impression of recovery, the participants will be asked the following: “Compared with the beginning of the first episode, how would you currently describe your back?” Positive scores represent recovery, and negative scores indicate a worsening of the symptoms. The scale will be applied before and after treatment and at the 4-month telephone follow-up.

#### Sit-to-stand test

Functional performance will be assessed through a sit-to-stand test. The patients will be instructed to sit and stand 5 times from a chair with a backrest with their arms crossed in front of them as quickly as they can [28]. The test will be timed, and immediately after the test, the patients will be questioned regarding the low back pain experienced during the test. The test will be performed during the first and last sessions prior to the application of the current and after 30 minutes of current stimulation.

#### Pain temporal summation

Temporal summation (TS) will be induced by an analogue pressure algometer (FPK20, Wagner Instruments, Greenwich, CT, USA) with a circular metal tip measuring 0.79 cm^2^. The evaluator will be trained prior to the data collection. The area selected for TS analysis will be the site indicated as the lower pain threshold in the low back algometry in which three stimuli per second will be applied with a pressure of approximately 2 kg/s to determine the best value for use in the TS test. Next, 10 stimuli will be performed using the algometer on the selected region. Each TS stimulus will be maintained for 1 second before being released, and the stimuli will be spaced at 1-second intervals. A timer will be used to ensure that the intervals are respected and that the stimuli are maintained. Participants will be instructed to report pain using the NRS, which will be posted on the wall in front of them, at the first, fifth and tenth stimulus [29]. The TS assessment will be performed prior to initiating the treatment protocol and prior to current application during the last session. To prevent sensitisation interference from the previously performed pain pressure threshold assessment, the TS assessment will begin 2 minutes after the PPT assessment.

#### Conditioned pain modulation

A cold pressor test will be used to assess the activation of the conditioned pain modulation (CPM) [30]. The conditioned stimulus will involve the immersion of the lower limb on the ipsilateral side of the more painful lumbar region. In cases of bilateral pain, the subject will be instructed to report the most painful side [31]. If no consensus can be reached regarding the most painful side, the right leg will be used. The limb will be immersed in a bucket containing water and ice at 4°C, 3 cm above the lateral malleolus of the ankle. The low back pain intensity will be assessed after 20 seconds of immersion using the NRS. The PPT at the low-back algometry points will be recorded 30 seconds after immersion. After removing the limb from the water, the participants will be questioned regarding their foot pain according to the NRS. A CPM activation test will be performed on the first day of treatment prior to initiating the stimulation and on the last day of the session prior to applying the current so that no interference occurs in the CPM assessment immediately after stimulation.

#### Analgesic use

To assess the use of analgesics during the treatment, the assessor will complete a record listing the days of the week from the beginning of the treatment until the date of the last session to note the use of analgesics or anti-inflammatory drugs and their dosage during treatment [10]. With this information, any reduction in the use of medications during treatment can be assessed.

### Procedure

#### Randomisation

After assessment, the patients will be referred to the therapist responsible for the treatment, who will open the sealed envelope prior to initiating the treatment to determine in which group the patient will be included (Group IFC 1 kHz, Group IFC 4 kHz or the IFC Placebo Group). The randomisation of groups will be performed using a computer-generated, random-number list compiled by an investigator not involved in the patient recruitment or data collection. The group allocation will be concealed by printing the group allocation onto cards and sealing them in consecutively numbered opaque envelopes. The envelopes will be stored in a secure cabinet accessibly only to the allocation investigator and will be opened immediately prior to the intervention allocation.

#### Study-blinding assessment

Following the treatment and the follow-up assessments, the therapist will ask the assessor if she believes that the patient received interferential current or the placebo. Additionally, the study participants will answer the same questions justifying their answers. The answers to these questions will be recorded and used to measure the effectiveness of the study blinding [32].

#### Data analysis

Data will be double-entered and analysis will be performed by a blinded statistician. All statistical procedures will be conducted following the intention-to-treat principles. Initially, the descriptive statistics will be used for the studied variables. The data normalisation will be analysed through a visual inspection of the histograms. The between-group differences in the measurements of the primary and secondary outcomes will be compared via linear mixed models using the interaction terms “group versus time” with a Tukey’s post-hoc test. For the data analysis, the SPSS (statistical package for social sciences) version 19.0 for Windows and Microsoft Excel 2007 will be used. All tests will be performed assuming a significance level of p < 0.05.

#### Sample size calculation

To obtain the total number of participants in the present study, the sample size was calculated to detect a difference of 1 point in the pain intensity outcome as measured by the numerical rating scale (NRS) (Costa et al. 2008) with a standard deviation of 1.47 points [33]. A statistical power of 80%, a 5% alpha and a possible sample loss of up to 15% were considered. Therefore, 50 patients per group (150 in total) will be required (Minitab, v. 15, State College, PA).

## Discussion

The results of the present study will provide more accurate estimates of the therapeutic effects and parameters of interferential currents. The last systematic review on the subject [12] suggests that new studies need to be performed given the lack of high-quality research on the use of interferential current in patients with low back pain. This study can also provide information on the effects of the current on the pain mechanisms and the optimum carrier frequency for use in the analgesia of chronic, nonspecific low back pain. The results of the present study may also assist physical therapists in making clinical decisions based on a high-quality randomised controlled trial. An appropriate choice of therapy can increase the efficacy of low back pain treatments, thereby increasing patients’ satisfaction and reducing the social costs associated with these patients.

The strengths of the present study include a high quality design that supports strong clinical evidence. The use of a placebo group enables an analysis of the therapy’s effectiveness and aids in understanding the placebo effect. The study’s blinding assessment indicates the reliability of the assessor and patient blinding. The present study is the first to compare different IFC carrier frequencies in patients suffering from pain and to assess the long-term effects of the treatment.

The study’s limitations include the impossibility of completely blinding the therapist due to the nature of the intervention and the lack of a control group (undergoing no treatment) for comparison with the other groups.

## Abbreviations

ADLs: Activities of daily living; TENS: Transcutaneous electrical nerve stimulation; CNS: Central nervous system; IFC: Interferential current; AMF: Amplitude-modulated frequency; NRS: Numeric rating scale; PPT: Pressure pain threshold; ICC: Intraclass correlation coefficients; VAS: Visual analogue scale; TS: Temporal summation; CPM: Conditioned pain modulation.

## Competing interests

We certify that no party with a direct interest in the results of the research supporting this article has or will confer benefits on us or on any organisation with which we are associated.

## Authors’ contributions

JBC, REL, LOPC and KAS were responsible for conceiving and designing the study. REL and LPOC are the study coordinators. JBC and NTBO are responsible for data collection. All authors have contributed for writing and approved this manuscript.

## Pre-publication history

The pre-publication history for this paper can be accessed here:

http://www.biomedcentral.com/1471-2474/14/195/prepub
